# Designing a new cropping system for high productivity and sustainable water usage under climate change

**DOI:** 10.1038/srep41587

**Published:** 2017-02-03

**Authors:** Qingfeng Meng, Hongfei Wang, Peng Yan, Junxiao Pan, Dianjun Lu, Zhenling Cui, Fusuo Zhang, Xinping Chen

**Affiliations:** 1 College of Agronomy and Biotechnology, China Agricultural University, Beijing 100193, China; 2 Center for Resources, Environment and Food Security, China Agricultural University, Beijing 100193, China; 3 Key Laboratory of Tea Biology and Resources Utilization, Ministry of Agriculture, Tea Research Institute, Chinese Academy of Agricultural Sciences, Hangzhou 310008, China

## Abstract

The food supply is being increasingly challenged by climate change and water scarcity. However, incremental changes in traditional cropping systems have achieved only limited success in meeting these multiple challenges. In this study, we applied a systematic approach, using model simulation and data from two groups of field studies conducted in the North China Plain, to develop a new cropping system that improves yield and uses water in a sustainable manner. Due to significant warming, we identified a double-maize (M-M; *Zea mays* L.) cropping system that replaced the traditional winter wheat (*Triticum aestivum* L.) –summer maize system. The M-M system improved yield by 14–31% compared with the conventionally managed wheat-maize system, and achieved similar yield compared with the incrementally adapted wheat-maize system with the optimized cultivars, planting dates, planting density and water management. More importantly, water usage was lower in the M-M system than in the wheat-maize system, and the rate of water usage was sustainable (net groundwater usage was ≤150 mm yr^−1^). Our study indicated that systematic assessment of adaptation and cropping system scale have great potential to address the multiple food supply challenges under changing climatic conditions.

Agriculture faces rapidly growing challenges because it must supply food to an increasing population under shifting climate conditions^1^,^2^,^3^,^4^. Furthermore, water scarcity is expected to increasingly limit crop production in many areas, and some analyses suggest that it is already doing so^5^,^6^,^7^. To cope with these multiple challenges, researchers, policy makers, and farmers urgently need to consider different levels of agricultural adaptation^8^,^9^.

To counteract the negative effects of climate change, researchers have generally emphasized incremental adaptation to existing cropping systems, such as the adjustment of planting dates and the use of cultivars with longer growing periods^8^,^10^,^11^,^12^. Although these adaptations might indeed be effective in terms of improved grain yield, a growing sensitivity of crop production to water shortages has also been observed^13^,^14^. Considering the potential for further reductions in water availability for agriculture^15^, such incremental adjustments are unlikely to provide long-term solutions to the problems of inadequate food and water supplies^16^. Thus, more extensive changes in cropping systems should be considered.

The North China Plain (NCP), which is facing significant warming and water scarcity, has received worldwide attention^5^,^17^,^18^,^19^. The NCP provides more than one-fourth of the national food supply in China^20^. The dominant agricultural system in this region involves double cropping. Winter wheat (*Triticum aestivum* L.) is rotated with summer maize (*Zea mays* L.), resulting in two harvests annually^21^. To adapt for the changing climate, researchers have adjusted planting and harvesting dates, and identified cultivars that require more days to maturity, to improve grain yield^18^,^21^,^22^. However, because of the large gap between precipitation and water demand in the winter wheat–summer maize system, such adaption requires more than 250 mm groundwater for irrigation each year, even when water usage is optimized^23^. As a consequence, the groundwater table in the northern part of the NCP has declined by about 1 m yr^−1 ^24. This decline has caused a series of environmental problems and has restricted sustainable development in the region^17^. It is now clear that incremental adaptation of planting dates and crop cultivars is inadequate in this region. Rather than small adjustments to the current cropping system, more systematic changes are now required to meet the challenges of global warming, water scarcity, and a growing demand for food.

Although climate warming poses serious problems for agriculture, it also enables the usage of new cropping systems because of increased available growing degree days (GDD), which are a measure of heat accumulation^4^. Reports from some studies have described the possibility of using new cropping systems in response to global warming. For example, warming might allow irrigated maize in central Chile to increase from a single crop to a double crop annually in future climate scenarios^25^. Although this type of cropping system change could be important, it has received only limited theoretical attention and has seldom been tested experimentally.

In the current study, we tested the hypothesis that a new cropping system would increase yield and reduce water usage relative to the traditional winter wheat–summer maize system in the NCP. Maize, a C_4_ crop, could efficiently use water and solar radiation to achieve remarkably high and stable grain yields^26^,^27^. Therefore, we attempted to develop a new double-maize (M-M) system (with two maize crops annually) to replace the traditional winter wheat–summer maize system (with one C_3_ and one C_4_ crop annually) at the site of Quzhou in the NCP (Supplementary Fig. S1). To design the new system, we used the Hybrid-Maize model and historical climate data. We then evaluated different combinations of two seasons’ cultivars in terms of grain yield and water usage to establish the new system in a 2-year field experiment.

Finally, we conducted an experiment to compare the new M-M system with the conventional winter wheat–summer maize (Con.W-M) system, and the optimized winter wheat–summer maize (Opt.W-M) system with incremental adaptation over 2 years. We attempted to answer three questions: (1) Given the recent climate changes, could maize be double cropped each year in the NCP? (2) If the answer is “yes,” how should the M-M system be designed? and (3) How does the new M-M system perform compared with the winter wheat–summer maize system, with respect to grain yield and water usage?

## Results

### Climatic trends and design of the double-maize system

**Figure 1:**
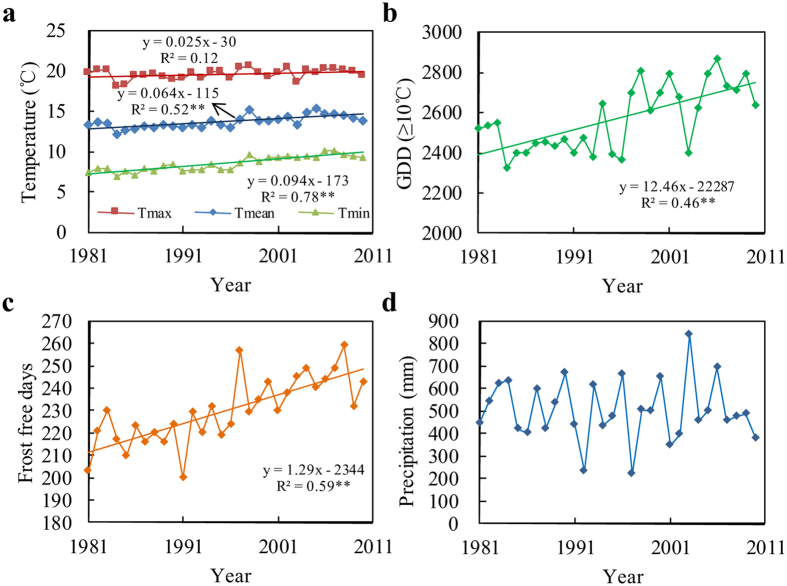
Changes in temperature, number of frost free days and precipitation from 1981 to 2010 at the Quzhou Experimental Station. (**a**) The annual maximum, mean, and minimum temperatures (Tmax, Tmean, and Tmin); (**b**) The number of available growing degree days; (**c**) The number of frost free days; and (**d**) Precipitation. ^**^P < 0.05.

From 1981 to 2010, Quzhou experienced significant warming. The mean temperature (T_mean_) increased by 0.64 °C decade^−1^. The minimum temperature (T_min_) increased by 0.94 °C decade^−1^ (Fig. 1a), while the maximum temperature (T_max_) did not change significantly. As a result, the annual GDD in the frost-free period increased significantly, by 12.5 °C yr^−1^ (Fig. 1b). Meanwhile, average length of the frost-free period increased by 1.3 days annually (Fig. 1c). The annual precipitation did not change significantly, and averaged 502 mm from 1981 to 2010 (Fig. 1d).

To test the feasibility of using the M-M system under conditions of significant warming, we used the Hybrid-Maize model and climate data from the recent decade (2001–2010) to simulate maize growth. We used the four combinations of long-season (LS) and short-season (SS) maize cultivars (SS-SS, SS-LS, LS-SS, and LS-LS; Table 1). Yield potential simulated by the Hybrid-Maize model was more than 19.0 Mg ha^−1^ for the first three treatments (SS-SS, SS-LS, and LS-SS). Yield potential was less than 19.0 Mg ha^−1^ for the LS-LS treatment, due to the severe risk of frost at the end of the summer maize season. The overall probability of frost occurrence during grain filling in the second season was 100% for all treatments except the SS-SS treatment.

### Grain yield and temporal match between maize growth and accumulation of growing degree days in field experiment I: double-maize system establishment

In field experiment I, grain yield was highest for the SS-LS treatment and similar among the other three treatments in 2012 and 2013 (Table 2). Grain yield of the SS-LS treatment was 20.3 Mg ha^−1^ yr^−1^ in 2012 and 17.5 Mg ha^−1^ yr^−1^ in 2013, which were 25–32% and 22–25% higher, respectively, than the yields of the other three treatments. The ratio between yield and yield potential ranged from 69% to 93% (Table 2). Among the four treatments, the highest proportion of the yield potential was achieved in the SS-LS treatment in 2012 (93%) and 2013 (88%), respectively. For all treatments, the ratio between yield and yield potential was higher for spring maize than for summer maize. The SS-LS treatment showed the greatest potential for wide demonstration to farmers, due to the higher grain yield and achievement of yield potential.

**Figure 2:**
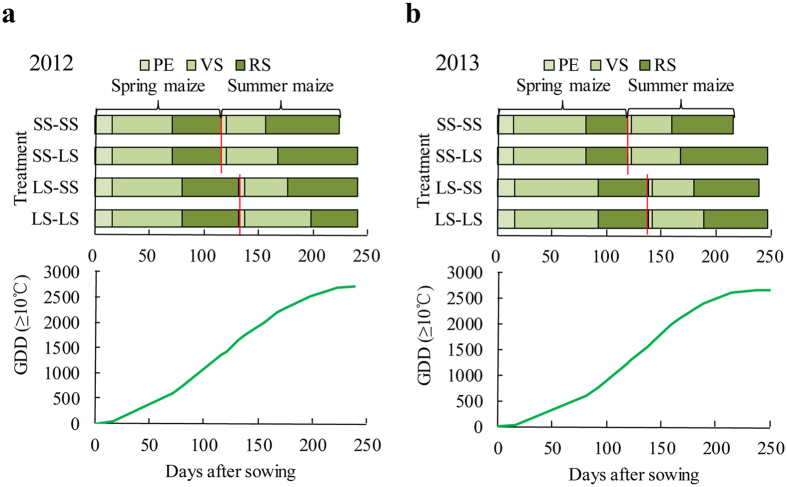
Duration of the pre-emergence stage (PE, from sowing to emergence), vegetative stage (VS, from emergence to silking), and reproductive stage (RS, from silking to physiological maturity), and the cumulative number of growing degree days after the spring maize sowing for the double-maize (M-M) system in field experiment I in 2012 (**a**) and 2013 (**b**). The treatments are explained in Table 1.

In 2012, the spring maize yield was similar among all four treatments (Table 2), but the summer maize yield was 91–129% higher for the SS-LS treatment than for the other treatments. The ability of the SS-LS treatment to achieve the highest grain yield could be explained, in part, by the temporal match between maize growth and GDD accumulation (Fig. 2). The SS cultivar increased the time available for summer maize, and the LS cultivar, when grown as summer maize, could grow longer before the onset of lower temperatures. In 2012, the summer maize growing season was 16 days longer for the SS-LS treatment than for the other treatments (Fig. 2a). For the SS-LS treatment, GDD accumulated slowly during the reproductive stage of summer maize, and the T_mean_ was 18.2 °C, which was suitable for grain filling. As a result, the reproductive stage for summer maize was 4–30 days longer for the SS-LS treatment than for the other treatments. In 2013, the SS-LS treatment achieved a high grain yield, also mainly from summer maize (Table 2), and the summer maize season was 19–31 days longer for this treatment than for the other treatments (Fig. 2b).

The high grain yield of the SS-LS treatment also resulted from tolerance of the cultivars to environmental stresses. When the SS cultivar Deimeiya 1 (DMY1) was planted as spring maize, it achieved 100% of its yield potential (Table 2). In contrast, the SS cultivar performed poorly when it was planted as summer maize in the SS-SS and LS-SS treatments (Table 2), mainly because this high-latitude cultivar could not tolerate the hot and wet summers in the NCP. For the LS-LS treatment, severe frost required harvest of summer maize before maturity, which reduced grain yield.

For all treatments, the annual yields were lower in 2013 than in 2012, due mainly to lower spring maize yields. These low yields were attributable to the occurrence of more days with rain and less solar radiation during the spring of 2013 (Supplementary Fig. S2).

### Irrigation, water use efficiency, and water balance in field experiment I: double-maize system establishment

For all treatments, total irrigation in 2012 was 140 mm, which was used entirely for the spring maize crop (Table 3). In 2013, total irrigation was 240 mm, of which 150 mm was used for the spring maize crop and 90 mm was used for the summer maize crop. Irrigation differed between the years because the precipitation differed (Supplementary Fig. S2). Precipitation for the entire season was 504–517 mm in 2012, but only 437–447 mm in 2013 (Table 3). Most differences in precipitation occurred during the summer maize season. Depending on the treatment, precipitation for summer maize ranged from 230 to 249 mm in 2012 and from 114 to 163 mm in 2013.

Calculated evapotranspiration (ET) for the four treatments ranged from 576 to 643 mm in 2012 and from 619 to 673 mm in 2013. Water use efficiency (WUE) of the SS-LS treatment averaged 2.93 kg m^−3^ in both years (Table 3), which was 21–35% and 13–20% higher than WUE for the other treatments in 2012 and 2013, respectively.

The calculated net groundwater usage ranged from 77 to 79 mm in 2012, and averaged 167 mm for all treatments in 2013 (Table 3). In 2012, groundwater usage was 99–101 mm during the spring maize season, and groundwater was partially recharged during the summer maize season. In 2013, groundwater usage was 101–105 mm for spring maize and 62–66 mm for summer maize. For the SS-LS treatment, annual net groundwater usage was 77 mm yr^−1^ in 2012 and 167 mm yr^−1^ in 2013.

### Grain yield in field experiment II: cropping system comparison

**Figure 3:**
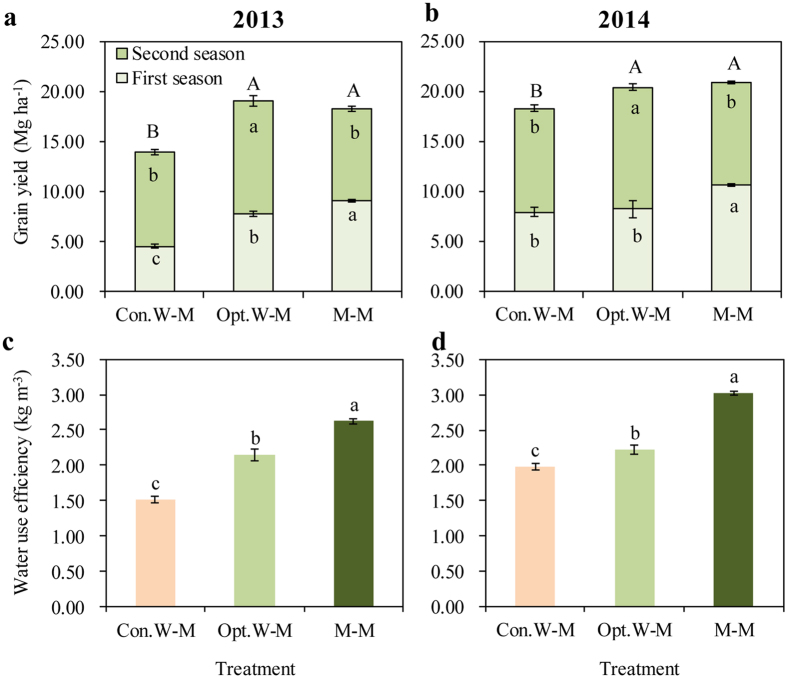
Grain yield and water use efficiency for the conventional winter wheat–summer maize system (Con.W-M), optimized winter wheat–summer maize system (Opt.W-M) and double-maize system (M-M) in the field experiment II in 2013 and 2014. For both Con.W.M and Opt.W-M treatments, first season was wheat and second season was maize. For M-M treatment, both seasons were maize. In (**a**) and (**b**), for the same season, three treatments means followed by the same letter are not significantly different at *P* < 0.05; the lowercases showed the compassion for the first and second season; the capitals showed the compassion for the sum of first and second season; bars represent standard error. In (**c**) and (**d**), three treatments means followed by the same letter are not significantly different at *P* < 0.05. Bars represent standard error.

In both years, the M-M treatment significantly improved grain yield compared with the Con.W-M treatment, which was based on current farmers’ practices, and achieved yield similar to that of the Opt.W-M treatment (Fig. 3). Grain yields of the M-M treatment were 18.3 Mg ha^−1^ in 2013 and 20.9 Mg ha^−1^ in 2014, which were 31% and 14% higher, respectively, than the yields of the Con.W-M treatment.

Grain yield of the M-M treatment was improved mainly in the first maize season compared with winter wheat (Fig. 3). In 2013, maize yield of the M-M treatment during the first season was 9.1 Mg ha^−1^, which was 98% and 17% higher than the wheat yields of the Con.W-M and Opt.W-M treatments, respectively. For the second maize season, grain yield of the M-M treatment was 9.2 Mg ha^−1^, which was similar to the maize yield of the Con.W-M treatment and 19% lower than the maize yield of the Opt.W-M treatment. In 2014, maize yield of the M-M treatment in the first season was 10.7 Mg ha^−1^, which was 29% and 35% higher than the wheat yields of the Con.W-M and Opt.W-M treatments, respectively. Similar to 2013, the yield for the second maize crop of the M-M treatment in 2014 was similar to the yield of the Con.W-M treatment, and 16% lower than the maize yield of the Opt.W-M treatment.

### Irrigation, water use efficiency, and water balance in field experiment II: cropping system comparison

In 2013, irrigation for the M-M treatment was 155 mm less than that for the Con.W-M treatment and 70 mm less than that for the Opt.W-M treatment, with optimized water management (Table 4). Compared with the Con.W-M and Opt.W-M treatments, the M-M treatment achieved more than 70% reduction of water usage during the first crop season. Irrigation water usage during the wheat season was 255 mm for the Con.W-M treatment and 180 mm for the Opt.W-M treatment, which were 125 mm and 50 mm, respectively, greater than that for the M-M treatment in the spring maize season. Similar trends were observed in 2014.

In both years, the lowest ET was observed for the M-M treatment (Table 4). In 2013, ET of the M-M treatment averaged 589 mm, which was 24% and 21% lower than the Con.W-M (779 mm) and Opt.W-M (750 mm) treatments, respectively. ET of the Con.W-M and Opt.W-M treatments did not differ significantly. Similar results for ET were observed in 2014. The highest WUE was observed in the M-M treatment during both years (Fig. 3). The WUE averaged 2.62 kg m^−3^ for the M-M treatment in 2013, which was 74% and 22% higher than for the Con.W-M and Opt.W-M treatments, respectively. The WUE averaged 3.02 kg m^−3^ for the M-M treatment in 2014, which was 53% and 36% higher than for the Con.W-M and Opt.W-M treatments, respectively.

The calculated net annual groundwater usage averaged 275 mm for the Con.W-M treatment and 208 mm for the Opt.W-M treatment (Table 4). In contrast, groundwater usage averaged 139 mm for the M-M treatment over 2 years. For all treatments, more than 70% of groundwater usage occurred during the first crop season. In 2013, 80% and 77% of groundwater usage occurred during the wheat season for the Con.W-M and Opt.W-M treatments, respectively. In 2014, more than 81% of groundwater usage occurred during the wheat season for the Con.W-M and Opt.W-M treatments.

## Discussion

Food supply in the NCP, an important agricultural area in the world, is being increasingly challenged by climate change and water scarcity, and has received increasing global attention^5^,^17^. Several studies have focused on improving grain yield to offset the negative effects of climate change on crop production^18^,^21^ and to reduce water usage through cropping system innovation and improved agronomic practices^24^. The challenge has been to leverage cropping system innovation and improved agronomic practices to improve grain yield and water usage.

In this study, we examined whether a change from the Con.W-M system to the M-M system could achieve high productivity and reduce water usage in the NCP. Grain yield in our M-M system averaged 19.6 Mg ha^−1^ yr^−1^ over 2 years, which was 21% higher than that of the Con.W-M system and similar to that of the Opt.W-M system (Fig. 3). Irrigation in our M-M system averaged 190 mm, which was 155 mm lower than for the Con.W-M system and 79 mm lower than for the Opt.W-M system (Table 4). The WUE of the M-M system increased by 22–74% (Table 4). Most importantly, groundwater usage was substantially lower for the M-M system (139 mm yr^−1^) than for the Con.W-M (275 mm yr^−1^) and Opt.W-M (208 mm yr^−1^) systems (Table 4). According to Chen *et al*.^28^, the decline in the NCP groundwater table could be avoided if regional recharging continues at its current rate and groundwater exploitation is ≤150 mm yr^−1^. Our findings suggest that the new M-M system could achieve high productivity with sustainable water usage.

In contrast to the extensive adaptations presented in the current study, most previous adaptations of cropping systems in response to climate warming and water scarcity have been incremental^8^,^9^,^16^,^29^. For example, a typical incremental adaptation to climate change in the NCP is to adjust wheat planting and maize harvesting, and to grow LS maize cultivars. Over the last 40 years, this incremental adaptation has increased annual grain yield in the NCP by 4–6%^21^. Although this is an adaptation to adverse weather, it fails to reduce water usage (Table 4). Because of the large gap between precipitation and water demand, the traditional system consumes more than 200 mm groundwater annually in farmers’ fields (Table 4).

To reduce the demand for water in the NCP, several research teams have attempted to develop new, lower-intensity cropping systems^23^,^30^. For example, a 6-year field study showed that systems with winter wheat and summer maize in the first year and spring maize alone (maize monoculture) in the second year could substantially reduce irrigation and groundwater usage in the NCP^23^. However, grain yields of these systems with increased fallow periods were 16–31% lower than the yield of the traditional winter wheat–summer maize system.

The higher grain yield in our M-M system compared with the conventional system could be explained, in part, by the cultivation of two, rather than one, C_4_ crops annually. Maize more effectively uses solar radiation and other resources, and, thus has a higher yield potential, than a C_3_ crop such as wheat. Higher grain yield in the new system could also be explained by the temporal match between maize growth and available resources, including GDD and light. For the new system, yield was higher for the SS-LS treatment than for the other three treatments in experiment I in 2012 and 2013, due to the short duration of the SS cultivar, as spring maize provided a long and optimal growing environment for summer maize (Fig. 2). The higher grain yield in the new system could also be explained by the tolerance of the cultivars to sources of stress in the hot and wet environment of the NCP during the summer. The SS cultivar DMY1, which was bred at high latitudes, is adapted to cool temperatures and performs well in the spring, but not in the summer (Table 2). Other studies have shown that SS cultivars are useful for dealing with the increased risk of drought, which is often associated with climate change^31^. The development of cultivars that are tolerant to the abiotic and biotic stresses likely to be encountered with climate change in specific regions would be useful^32^.

The M-M system reduced irrigation, increased WUE, and reduced groundwater consumption, which could also be explained by growing two, rather than one, C_4_ crops (maize) annually. WUE is known to be greater for C_4_ cereals than for C_3_ cereals^26^. In addition, the demand for water and the occurrence of precipitation coincided temporally in the new system (Table 4). More than 90% of precipitation occurred during the maize growth period in the M-M system (Supplementary Fig. S2). The annual water requirements for the M-M system in experiment II were 589 mm in 2013 and 585 mm in 2014 (Table 4). Thus, the gaps between water supplied by precipitation and water demand by the M-M system were 155 mm in 2013 and 214 mm in 2014.

Accounting for recharge through irrigation and precipitation, net annual groundwater consumption for the M-M system averaged 139 mm. However, precipitation (434 mm) was 68 mm lower in 2013 and 131 mm lower in 2014 than the 30-year average from 1981 to 2010. When we use the 30-year average rather than the 2-year average in our calculations, estimated groundwater consumption is less than 139 mm. For the traditional winter wheat–summer maize system, in contrast, only 20–30% of the total precipitation occurs during the winter wheat growing season (Table 4)^33^,^34^,^35^. To achieve a high grain yield of wheat, more than 400 mm yr^−1^ supplementary irrigation is usually applied in some areas of the NCP^24^,^36^.

In this study, we used plastic film to increase the total available GDD in the spring maize season for the M-M system. Plastic film also could influence the soil-crop water process by changing the balance between evaporation and transpiration, especially during the early maize growth stages. For example, some studies have shown that plastic film reduced soil evaporation by 60–80 mm^37^. However, total ET during the whole maize season did not change significantly because of the increased transpiration under plastic film condition with larger biomass^38^. Plastic film could effectively reduce soil evaporation by creating an impermeable barrier, increasing canopy transpiration and thereby enhancing grain yield and WUE^38^. Our M-M system, as a systematic adaptation, involved not only biological understanding together with crop modeling (i.e., regarding cultivars and planting dates used), but also novel management techniques (e.g., used of plastic film, water management).

In the NCP, the warming trend is predicted to continue^39^ and water scarcity could become more severe^6^ in the future. While traditional adaptation of existing crop systems addresses these challenges to a limited extent, we followed a systematic adaptation approach to develop new cropping systems that achieved high productivity and sustainable water usage under changing climate conditions. We found the M-M system to be appropriate for the NCP in the face of significant warming, as it achieved high productivity and reduced groundwater consumption. This type of systematic adaptation could be usefully applied in other areas where climate change and water shortages significantly affect agricultural productivity.

## Methods

### Site description

The NCP is located in central-eastern China and includes Beijing, Tianjin, Hebei, Shandong, Henan, Anhui, and Jiangsu provinces (Supplementary Fig. S1). The study site was the Quzhou Experimental Station, China Agricultural University (115.0°E, 36.5°N, 40 m above sea level), in Quzhou County, Heibei Province (Supplementary Fig. S1). Quzhou County is an area of intensive agriculture and is typical of the NCP, in that more than 80% of the agricultural fields in the county are used for winter wheat and summer maize in annual rotation. The current winter wheat and summer maize system at Quzhou is highly productive, with grain yields as high as 14.9 Mg ha^−1^ yr^−1 ^23.

This region has a typical warm-temperate, sub-humid, continental monsoon climate, with hot, rainy summers and cold, dry winters. The annual T_mean_ is 13.2 °C, and annual precipitation is about 500 mm. Approximately 70–80% of the total precipitation occurs during the summer maize growing season^33^,^34^,^35^, and most irrigation is applied during the winter wheat growing season.

At the Quzhou site, the soil texture is clay loam, with a bulk density of 1.36 g cm^−3^ for the 0–30-cm soil layer. The chemical properties of the 0–30-cm soil layer were as follows: organic matter content, 14.2 g kg^−1^; total N, 0.83 g kg^−1^; Olsen-P, 7.2 mg kg^−1^; NH_4_OAc-K, 125 mg kg^−1^; and pH, 8.3.

### Climate data

Data for temperature, including daily T_max_, T_mean_, and T_min_, precipitation, sunshine hours, wind speed, and relative humidity from 1981 to 2013 were obtained from the Quzhou Meteorological Station. Daily solar radiation was estimated by sunshine hours according to Jones^40^.

### Model-based cropping system design

We used the Hybrid-Maize model developed by the University of Nebraska-Lincoln (USA) and recent climate data (2001–2010) to investigate the feasibility of an M-M system and to identify the appropriate combination of cultivars and planting times at the Quzhou Experimental Station. The Hybrid-Maize model combines the strengths of CERES-Maize models and assimilate-driven generic crop models, such as SUCROS and WOFOST^41^,^42^. The Hybrid-Maize model could simulate yield under optimized water and rainfed conditions. It could simulate the daily development and growth of maize. This model has been tested and used widely to predict maize production in USA^43^, South Asia^44^, and China^14^,^45^,^46^,^47^. Previous studies have shown that the model performs well in a variety of regions, including the Quzhou site^48^. To simulate grain yield, the model requires data for daily total solar radiation, T_max_ and T_min_, and ET. Other model inputs included cultivar GDD (to maturity), date of planting, and plant population density.

Zhengdan 958 (ZD958) is a commonly planted LS maize cultivar grown in the NCP that requires 1719 GDD to mature. The total available frost-free GDD averaged 2704 from 2001 to 2010 at the Quzhou site (Fig. 1b). Although a warming climate increased available GDD at the Quzhou Station from 1981 to 2010, the increases could not support the double cropping of ZD958 in a single year. Therefore, we introduced the SS cultivar DMY1, which was developed in Heilongjiang Province, China, for cropping at high latitudes (>47°N). DMY1 requires only 1383 GDD to mature. The double cropping of ZD958 and DMY1 would require a total of 3102 GDD, which is 398 GDD more than provided by local conditions. To increase the number of GDD provided, we considered the usage of plastic film mulch^49^ for the first crop season. Recently, a new module of plastic film mulch was developed for the Hybrid-Maize model^50^. We used a plant population of 75,000 ha^−1^ for all simulations, and we calculated grain yield based on 15.5% water content.

### Field experiment I: double-maize system establishment

For M-M system, the initial design comprised combinations of the SS cultivar (DMY1) and LS cultivar (ZD958; SS-LS and LS-SS). For comparison purposes, SS-SS and LS-LS combinations were also included in the design.

For the M-M system, the yields of four combinations of SS and LS maize cultivars were predicted using model simulations based on 2001–2010 climate data (Table 1). The simulations were then used to design a field experiment, which was conducted in 2012 and repeated in 2013 at the Quzhou site. A completely randomized block design was used with four treatments and four replicates (Table 1). Plots of 48 m^2^ (6 × 8 m) were separated by a 20-cm-wide buffer zone. Daily weather data for the growing seasons in both years are shown in Supplementary Fig. S2.

Spring maize was planted on March 18 in 2012 and 2013. Spring maize was harvested when mature, and summer maize was harvested when mature or when the temperature dropped below 0 °C. In each plot, maize was planted 5 cm deep and plants within rows were 22 cm apart. Rows were 60 cm apart.

Before spring maize was planted, a base fertilizer (100 kg N ha^−1^, 100 kg P_2_O_5_ ha^−1^, and 100 kg K_2_O ha^−1^) was broadcast and then turned over to place the fertilizer in the subsurface. Additional N fertilizer was side dressed at 80 kg N ha^−1^ at the 10-leaf stage. Before summer maize was planted, a basal fertilizer (100 kg N ha^−1^, 45 kg P_2_O_5_ ha^−1^, and 90 kg K_2_O ha^−1^) was applied, and an additional 80 kg N ha^−1^ was side dressed at the 10-leaf stage.

The irrigation schedule was based on field conditions and weather data at critical growth (six-leaf and silking) stages. At these stages, the soil-water quick test (alcohol burning method)^51^ was used to calculate the amount of irrigation water to apply. Optimized irrigation was used to keep the soil water content between 45% and 80% plant available water content. In 2013, 70 mm irrigation water was applied each at the six-leaf and silking stages for spring maize, and no irrigation was applied for summer maize. In 2014, 90 mm irrigation water at the six-leaf stage and 60 mm irrigation water at silking were applied for spring maize, and 90 mm irrigation water was applied at silking for summer maize. The plots were mulched with plastic film (0.7 m wide and 0.005 mm thick) to cover the soil surface during the first crop cycle of each year.

### Field experiment II: cropping system comparison

According to the results from experiment I, the best-performing treatment in terms of grain yield and water usage in both years was the SS-LS, which was selected as the M-M system. A randomized complete block design was employed with three treatments and four replications. The treatments included the M-M system, the Con.W-M system, based on farmers’ practices, and the Opt.W-M system, with incremental adaptations through integrated soil-crop system management. Cultivars, planting dates, planting density, and water management were optimized in the Opt.W-M system compared with the Con.W-M^52^. Crop development was designed to make maximum use of solar radiation and periods with favorable temperatures. Optimizing planting date would increase maize yield potential because of appropriate growth periods for grain filling under relatively lower temperatures; increasing density would also increase yield potential significantly by capturing more resources early during the growing season; and changing varieties with appropriate growth periods would increase maize yield potential by capturing more solar radiation and making use of favorable temperatures for growth. M-M system was according to field experiment I. Plots of 900 m^2^ (30 × 30 m) were separated by a 200-cm-wide zone. Daily weather data for the growing seasons in both years are also shown in Supplementary Fig. S2.

For the Con.W-M treatment, cultivars, planting date, and crop management followed current farmers’ practices. Winter wheat was planted on October 18 in 2013 and October 16 in 2014, and harvested in early June. Summer maize was planted immediately after the wheat harvest. The cultivars used in this study were LX99 for winter wheat and ZD958 for summer maize. Both cultivars are commonly grown in this region. The Con.W-M system was considered to be fully irrigated, following farmers’ current practices.

Depending on precipitation, the Con.W-M treatment was irrigated three to five times for wheat and one or two times for maize. The amount of irrigation water ranged from 60 to 100 mm, based on soil moisture and farmers’ practices. Nitrogen management for the Con.W-M treatment followed farmers’ practices in the NCP. Nitrogen input was 550 kg ha^−1^ yr^−1^, of which 300 kg ha^−1^ yr^−1^ was for winter wheat and 250 kg ha^−1^ yr^−1^ was for summer maize^53^.

For Opt.W-M treatment, planting date and crop management were adopted according to soil-crop system management^51^. Winter wheat was planted on October 6 in 2013 and 2014, and harvested in early June. Summer maize was planted immediately after the wheat harvest. The cultivars used in this treatment were the same as for the Con.W-M treatment. The rate and timing of irrigation in the Opt.W-M and M-M systems were determined according to soil water content tests performed at the beginning of critical growing seasons. Nitrogen management for the Opt.W-M treatment followed the optimized management practices. Nitrogen input was 390 kg ha^−1^ yr^−1^, of which 205 kg ha^−1^ yr^−1^ was for winter wheat and 185 kg ha^−1^ yr^−1^ was for summer maize.

At maturity, plants from a 7.2-m^2^ area in each plot in experiment I and a 150-m^2^ area in each plot in experiment II were selected for the measurement of grain yield. The selected plants were dried for 24 h at 75 °C to a constant weight. Grain yield was averaged over four replicates and adjusted to 15.5% water content. Before planting and at harvest of each crop, the soil water content in each plot was measured gravimetrically at 30-cm intervals, at 0–200 cm soil depth, except that the interval for the deepest layer was 20 cm. Soil moisture was recorded after oven drying at 105 °C for 24 h to a constant weight. Then, total soil water content was calculated by summing all soil moisture contents of the sampled layers in the 2-m soil profile.

### Data analyses

To detect temporal trends, 30 years of climate data (1981–2010) from Quzhou for the following variables were regressed against time: annual T_max_, T_mean_, T_min_, precipitation, calculated theoretical GDD, and number of days in the frost-free period. Student’s *t*-tests at 95% and 99% confidence levels were used to evaluate the slopes of the linear regression lines against time.

The GDD was estimated with the following formula^42^:


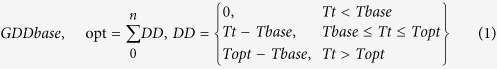


where *n* is the number of days, T_t_ is the daily average temperature, and T_opt_ is the optimum temperature for maize. T_base_ and T_opt_ were set at 10 °C and 34 °C, respectively^41^,^42^.

ET was calculated using the soil-water balance equation for the entire growing season and individual growth periods, as follows^54^:


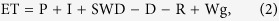


where ET is total ET for the growing season (mm), P is precipitation (mm), I is irrigation water quantity (mm), SWD is the change in soil water storage at the measured soil depth between planting and harvesting (mm), D is drainage below the root zone (mm), R is surface runoff (mm), and Wg is water used by the crop through capillary rise from groundwater (mm). R was ignored because no runoff occurred in the NCP^23^,^35^. Because the groundwater table was more than 20 m below the soil surface, and soil water extraction did not occur below 4 m, capillary rise was considered to be negligible^55^.

Thus, in this study, ET was computed by the following simplified equation:


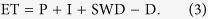


Drainage was estimated using a recharge coefficient (α) multiplied by the amount of irrigation and the effective rainfall (mm), as follows^23^,^35^:


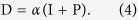


The recharge coefficient (α) depends on soil texture and the amount of irrigation or effective rainfall. The coefficient, applied to a large region and determined by the monitoring of changes in the groundwater table after irrigation, ranges from 0.1 for clay soil to 0.3 for sandy soil^56^. For the experimental conditions in this study, α was given values of 0.1 for irrigation or rainfall amounts less than 90 mm and 0.15 for irrigation or rainfall amounts between 90 and 120 mm.

Net groundwater usage was calculated as:


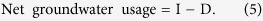


WUE (kg m^−3^) was calculated as:


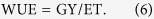


where GY is the grain yield (kg ha^−1^) and ET (mm) was calculated as in Eq. 3.

The effects of the treatments on the measured parameters in fields were evaluated by one-way analysis of variance using SAS software, and means were separated by the least significant difference at the 5% significance level (*P* < 0.05).

## Additional Information

**How to cite this article:** Meng, Q. *et al*. Designing a new cropping system for high productivity and sustainable water usage under climate change. *Sci. Rep.* **7**, 41587; doi: 10.1038/srep41587 (2017).

**Publisher's note:** Springer Nature remains neutral with regard to jurisdictional claims in published maps and institutional affiliations.

## Supplementary Material

Supplementary Information

## Figures and Tables

**Table 1:** Yield potentials for double-maize cropping systems (two maize crops per year) as simulated by the Hybrid-Maize model and as affected by the following four combinations of two maize cultivars.

Treatment	Crop	Sowing date	Maturity date	Yield potential per crop	CV	Yield potential per year	CV
		(month/day)	(month/day)	(Mg ha^−1^)	(%)	(Mg ha^−1^ yr^−1^)	(%)
SS-SS	Spring maize (DMY1)	3/18	7/13	9.1	35	21.7	19
	Summer maize (DMY1)	7/13	10/27	12.6	12		
SS-LS	Spring maize (DMY1)	3/18	7/13	9.1	35	21.5	20
	Summer maize (ZD958)	7/13	11/20*	12.4	13		
LS-SS	Spring maize (ZD958)	3/18	8/2	12.1	36	19.3	28
	Summer maize (DMY1)	8/2	11/20*	7.2	21		
LS-LS	Spring maize (ZD958)	3/18	8/2	12.1	36	16.5	36
	Summer maize (ZD958)	8/2	11/20*	4.4	52		

SS-SS, the short-season (SS) variety (DMY1) followed by the SS variety (DMY1). SS-LS, the SS variety (DMY1) followed by the LS variety (ZD958). LS-SS, the LS variety (ZD958) followed by the SS variety (DMY1). LS-LS, the LS variety (ZD958) followed by the LS variety (ZD958).

The simulations used climate data from 2001 to 2010. CV, coefficient of variation.

^*^The maize could not attain physiological maturity because of frost on this date. Overall probability of frost occurrence during grain filling (%): 100.

**Table 2:** Grain yield, yield potential, and the grain yield expressed as a percentage of yield potential for the double-maize system in the field experiment I in 2012 and 2013.

Year	Treatment	Spring maize			Summer maize			Both seasons		
		Grain yield	Yield potential	Grain yield/ Yield potential	Grain yield	Yield potential	Grain yield/ Yield potential	Grain yield	Yield potential	Grain yield/ Yield potential
		(Mg ha^−1^)	(Mg ha^−1^)	(%)	(Mg ha^−1^)	(Mg ha^−1^)	(%)	(Mg ha^−1^ yr^−1^)	(Mg ha^−1^ yr^−1^)	(%)
2012	SS-SS	10.1a	10.1	100	5.3b	12.2	43	15.4b	22.2	69
	SS-LS	10.2a	10.1	100	10.1a	11.7	86	20.3a	21.9	93
	LS-SS	11.1a	12.7	88	5.2b	9.0	58	16.3b	21.6	75
	LS-LS	11.1a	12.7	88	4.4c	6.1	72	15.5b	18.8	82
2013	SS-SS	7.9b	7.7	102	6.1b	10.6	58	14.0b	18.4	76
	SS-LS	7.9b	7.7	102	9.7a	12.1	80	17.5a	19.8	88
	LS-SS	8.8a	9.8	90	5.3c	8.1	65	14.1b	18.0	78
	LS-LS	9.1a	9.8	92	5.2c	6.7	78	14.3b	16.5	87

Treatments are described in Table 1. Within a column and year, means for grain yield followed by the same letter are not significantly different at *P* < 0.05.

Yield potential was simulated with 2012 and 2013 weather data at the Quzhou Experimental Station; Grain yield/Yield potential: Grain yield divided by yield potential* 100.

**Table 3:** Precipitation, irrigation, evapotranspiration (ET), water use efficiency (WUE), and net groundwater use for the double-maize system in the field experiment I in 2012 and 2013.

Year	Season	Treatment	Precipitation	Irrigation	ET	WUE	Net groundwater use
			(mm)	(mm)	(mm)	(kg m^−3^)	(mm)
2012	Spring maize	SS-SS	268	140	301a	3.45a	101
		SS-LS	268	140	347a	3.10a	101
		LS-SS	286	140	365a	3.08a	99
		LS-LS	286	140	386a	2.93a	99
	Summer maize	SS-SS	237	0	275ab	1.93bc	−23
		SS-LS	249	0	284a	3.56a	−24
		LS-SS	230	0	246c	2.13b	−22
		LS-LS	230	0	257bc	1.73c	−22
	Both seasons	SS-SS	504	140	576a	2.69b	79
		SS-LS	517	140	631a	3.25a	77
		LS-SS	517	140	611a	2.68b	77
		LS-LS	517	140	643a	2.41b	77
2013	Spring maize	SS-SS	285	150	307b	2.57ab	105
		SS-LS	285	150	293b	2.71a	105
		LS-SS	333	150	388a	2.28b	101
		LS-LS	333	150	366a	2.47b	101
	Summer maize	SS-SS	152	90	312b	1.97b	63
		SS-LS	163	90	379a	2.55a	62
		LS-SS	114	90	284b	1.86b	66
		LS-LS	114	90	273b	1.92b	66
	Both seasons	SS-SS	437	240	619b	2.27b	167
		SS-LS	447	240	673a	2.61a	167
		LS-SS	447	240	672a	2.10b	167
		LS-LS	447	240	639ab	2.24b	167

Treatments are described in Table 1. Within a column, year, and season, means followed by the same letter are not significantly different at *P* < 0.05.

**Table 4:** Precipitation, irrigation, evapotranspiration (ET), and net gr 686 oundwater use for per season crop in the conventional winter 687 wheat–summer maize system (Con.W-M), optimized winter wheat–summer maize system (Opt.W-M) and double-maize system (M-M) 688 in the field experiment II in 2013 and 2014.

Year	Season	Crop	Treatment	Precipitation	Irrigation	ET	Net groundwater use
				(mm)	(mm)	(mm)	(mm)
2013	First season	Wheat	Con.W/M	149	255	408a	218
		Wheat	Opt.W/M	149	180	418a	150
		Maize	M-M	273	130	243b	95
	Second season	Maize	Con.W/M	330	90	372a	55
		Maize	Opt.W/M	330	80	332a	46
		Maize	M-M	161	60	346a	41
	Both seasons	Wheat + maize	Con.W/M	479	345	779a	272
		Wheat + maize	Opt.W/M	479	260	750a	196
		Maize + maize	M-M	434	190	589b	136
2014	First Season	Wheat	Con.W/M	113	263	437a	228
		Wheat	Opt.W/M	113	208	390a	178
		Maize	M-M	205	130	339b	101
	Second season	Maize	Con.W/M	274	80	344a	50
		Maize	Opt.W/M	274	70	387a	41
		Maize	M-M	166	60	245b	41
	Both seasons	Wheat + maize	Con.W/M	387	345	781a	278
		Wheat + maize	Opt.W/M	387	278	778a	219
		Maize + maize	M-M	371	190	585b	141
