# Supplementary material for: Designing a new cropping system for high productivity and sustainable water usage under climate change
Source: Sci Rep. 2017 Feb 3;7:41587. doi: 10.1038/srep41587 (PMC5290742; doi:10.1038/srep41587)
Supplement: Supplementary Information [file srep41587-s1.docx]

**Designing a new cropping system for high productivity and sustainable water usage under climate change**

Qingfeng Meng^1,2^*, Hongfei Wang^2^*, Peng Yan^2,3^*, Junxiao Pan^2^, Dianjun Lu^2^, Zhenling Cui^2^, Fusuo Zhang^2^ & Xinping Chen^2^

^1^College of Agronomy and Biotechnology, China Agricultural University, Beijing100193, China, ^2^Center for Resources, Environment and Food Security, China Agricultural University, Beijing100193, China, ^3^Key Laboratory of Tea Biology and Resources Utilization, Ministry of Agriculture, Tea Research Institute, Chinese Academy of Agricultural Sciences, Hangzhou 310008, China.

* These authors contributed equally to this work.

Correspondence and requests for materials should be addressed to X.C. (email: chenxp@cau.edu.cn)


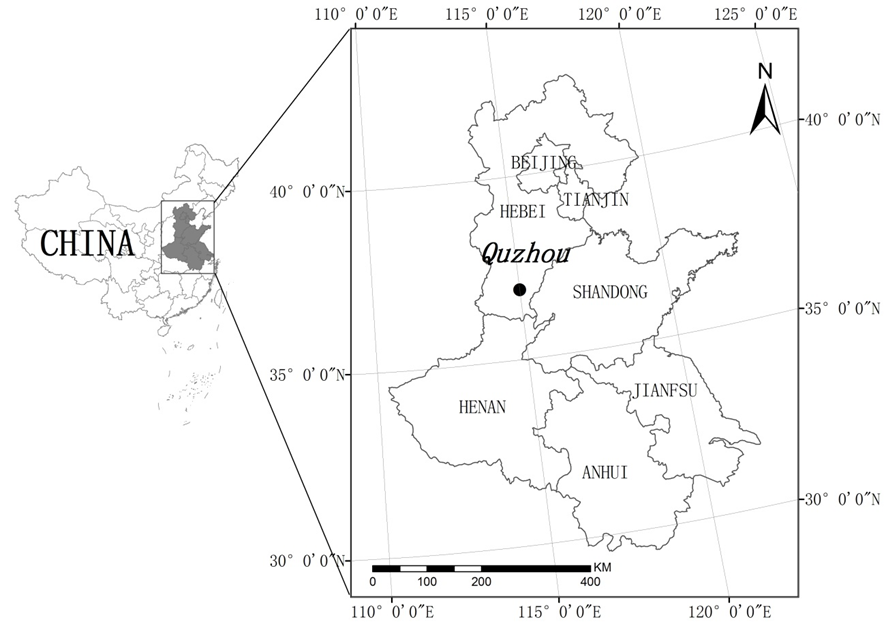
**Supplementary Figure S1. The location of the North China Plain (NCP) and the Quzhou experimental site in China.** This figure was made with ArcGIS 10.0 (https://www.arcgis.com/features/).

**Supplementary Figure S2. Daily precipitaion, mean temprature, and solar radiation in 2012, 2013 and 2014 at the Quzhou experimental site.**
